# Molecular detection of Merkel cell polyomavirus in basal cell carcinoma and perilesional tissue: a cross-sectional study^☆^^☆☆^

**DOI:** 10.1016/j.abd.2019.10.007

**Published:** 2020-05-11

**Authors:** Marianna Tavares Venceslau Gonçalves, Rafael Brandão Varella, Núbia Karla de Oliveira Almeida, Maria Angelica Arpon Marandino Guimarães, Flávio Barbosa Luz

**Affiliations:** a Department of Preventive Medicine, Hospital Universitário Clementino Fraga Filho, Universidade Federal do Rio de Janeiro, Rio de Janeiro, RJ, Brazil; b Department of Microbiology and Parasitology, Instituto Biomédico, Universidade Federal Fluminense, Rio de Janeiro, RJ, Brazil; c Department of Statistics, Universidade Federal Fluminense, Rio de Janeiro, RJ, Brazil; d Department of Dermatology, Hospital Universitário Antônio Pedro, Universidade Federal Fluminense, Rio de Janeiro, RJ, Brazil

Dear Editor,

The first and so far only oncogenic human polyomavirus, Merkel cell polyomavirus (MCPyV), was discovered in the last decade, infecting about 80% of Merkel cell carcinomas (MCC)^1^ and 40% of healthy individuals.^2^ Originated from cells located in the basal layer of the skin's epidermis, MCC is a rare and extremely aggressive type of non-melanoma skin cancer (NMSC), which generally affects the elderly, Caucasians and the immunocompromised in body areas with high UV exposure. Comprising the most prevalent neoplasia worldwide, NMSCs have been investigated to look for and elucidate other risk factors, such as infections by oncoviruses. The present authors’ group was responsible for the first study in a Brazilian population, with 32% MCPyV positivity in biopsies of various NMSCs.^3^ The present study, however, focused on investigating MCPyV exclusively in basal cell carcinoma (BCC) patients.

A convenience sampling composed of fresh-frozen biopsies and perilesional skin was analyzed, deriving from 35 consecutive patients with histopathological diagnosis of BCC treated at Antônio Pedro University Hospital (HUAP-UFF) between September 2017 and December 2018. Demographic data was collected during medical exam interview. Ethnicity was defined by the dermatologist according to patient's phototype, classified as “white” or “non-white.” Tumor location was used to infer solar exposure (high, moderate, or low). All samples had DNA extracted utilizing a commercial kit according to manufacturer instructions. MCPyV DNA was detected by nested polymerase chain reaction (PCR) for the LT3 region.^3^ Statistical analysis was performed using SPSS v. 20 software (SPSS Inc. – Chicago, IL, United States). All individuals agreed to participate by signing an informed consent. This study was approved by the University's Ethics Committee.

Eight of the 35 patients had more than one lesion. In general, patients were elderly (72.4 ± 11.8 years), predominantly female (62.9%), white (94.3%), and immunocompetent (97.1%). The most common comorbidities found were systemic arterial hypertension (68.6%) and diabetes mellitus type 2 (14.3%). Eleven patients with previous history of neoplasia and three with premalignant lesions at the time of surgery were also identified. The lesions were located mainly on the head (68.7%), neck (14.6%), upper limbs (10.4%), and lower limbs (6.3%).

Patients were classified as MCPyV + if at least one lesion had detectable viral DNA, resulting in nine (25.7%) MCPyV + patients. In regard to surgical margins, eight (22.8%) patients were MCPyV + . No statistically significant association (*p*-value ≤ 0.05) was found between MCPyV positivity and the gender, ethnicity, age, solar exposure, and immune status of the patients. The positivity for MCPyV when considering lesions (*n* = 45) and associated perilesional tissue was 45.5% and 54.5%, respectively. However, the level of agreement between lesion *vs.* associated perilesional tissue was considered low (kappa = 0.174) (Table 1), with ten (28.6%) patients showing disagreement for both samples.

**Table 1:** Agreement of MCPyV detection in BCC lesions (*n* = 45) and correlated perilesional skin.

Sample type		Perilesional skin		Kappa index (95% CI)
		+	–	
BCC	+	5	6	0.174 (−0.128 to 0.476)
	–	9	25	

BCC, basal cell carcinoma; MCPyV, Merkel cell polyomavirus.

As a recently discovered epitheliotropic and oncogenic virus, MCPyV became a sought-after research target in relation to NMSC samples. Since the important risk factors and etiology of NMSC still lack comprehensive understanding, this study aimed to evaluate MCPyV positivity in BCC, the most prevalent type of NMSC, in 35 patients. The results showed that 25.7% of patients were MCPyV positive, which is comparable with other studied populations.^3^

According to the results, BCC lesions and perilesional skin presented similar detection rates for MCPyV detection, which indicates that the virus is spread around the tissue regardless of the presence or absence of lesion, as indicated by the low level of agreement for viral detection. Whether MCPyV is incidental in this type of neoplasm or it plays a role in BCC development, through DNA integration and inhibition of cellular regulatory pathways,^4^ is not yet known. However, it is hypothesized that oncoviruses may provide the initial favorable conditions for the development of neoplasia by the “hit and run” mechanism, making it difficult to detect the agent once the lesion is established.^5^

The lack of association between viral detection in BCC and immunological status, age, ethnicity, and solar exposure indicate that they are not determinant of MCPyV in this type of neoplasm. Regarding patients’ comorbidities, none showed significance for MCPyV positivity (data not shown). Unfortunately, it was not possible to test these pre-malignant lesions for viral detection.

From these findings MCPyV appears not to correlate with major risk factors for BCC development; however, this study expands the knowledge of MCPyV in the Brazilian population, which is currently scarce. Even with its high prevalence of mixed race, the Brazilian population remains one of the most afflicted by NMSC worldwide and therefore all knowledge of possible risk factor associations is relevant to the country's public health.

## Financial support

This study was funded by FUNADERM (Fundo de Apoio à Dermatologia). This study was also partially funded by FAPERJ (Fundação de Pesquisa do Rio de Janeiro) and Coordenação de Aperfeiçoamento de Pessoal de Nível Superior (CAPES) – Financial Code 001.

## Authors’ contributions

Marianna Tavares Venceslau Gonçalves: Drafting and editing of the manuscript; collection, analysis, and interpretation of data; critical review of the literature; critical review of the manuscript.

Rafael Brandao Varella: Statistical analysis; approval of the final version of the manuscript; conception and planning of the study; drafting and editing of the manuscript; collection, analysis, and interpretation of data; participation in design of the study; critical review of the literature; critical review of the manuscript.

Núbia Karla de Oliveira Almeida: Collection, analysis, and interpretation of data; critical review of the literature, critical review of the manuscript.

Maria Angelica Arpon Marandino Guimarães: Drafting and editing of the manuscript; participation in design of the study; critical review of the manuscript.

Flávio Barbosa Luz: Approval of final version of the manuscript; conception and planning of the study; drafting and editing of the manuscript; collection, analysis, and interpretation of data; participation in design of the study; intellectual participation in the propaedeutic and/or therapeutic conduct of the studied cases; critical review of the manuscript.

## Conflicts of interest

None declared.

## References

[1] Feng H., Shuda M., Chang Y., Moore P. (2008). Clonal integration of a polyomavirus in human Merkel cell carcinoma. Science.

[2] Schowalter R.M., Pastrana D.V., Pumphrey K.A., Moyer A.L., Buck C.B. (2010). Merkel cell polyomavirus and two previously unknown polyomaviruses are chronically shed from human skin. Cell Host Microbe.

[3] Bellott T., Baez C., Almeida S.G., Venceslau M.T., Zalis M.G., Guimarães M.A. (2017). Molecular prevalence of Merkel cell polyomavirus in nonmelanoma skin cancer in a Brazilian population. Clin Exp Dermatol.

[4] Bofill-Mas S., Rodriguez-Manzano J., Calgua B., Carratala A., Girones R. (2010). Newly described human polyomaviruses Merkel Cell, KI and WU are present in urban sewage and may represent potential environmental contaminants. Virol J.

[5] Houben R., Grimm J., Willmes C., Weinkam R., Becker J., Schrama D. (2012). Merkel cell carcinoma and Merkel cell polyomavirus: evidence for hit-and-run oncogenesis. J Invest Dermatol.

